# A case report of anticonvulsive effects of CBD under ECT treatment

**DOI:** 10.1192/j.eurpsy.2025.2132

**Published:** 2025-08-26

**Authors:** M. Sánchez López, A. de Arriba Arnau, A. C. González Pezoa, R. Obrador Font, M. Urretavizcaya Sarachaga

**Affiliations:** 1 Psychiatry, Hospital Universitari de Bellvitge; 2 Universitat de Barcelona, Barcelona, Spain

## Abstract

**Introduction:**

Cannabidiol (CBD) is a non-psychoactive cannabinoid with therapeutic potential in various fields, such as its effect on mental health or antiepileptogenic, among others. For this reason, the interest in research on the potential effects of this substance is booming and the sale and consumption by the general population is increasing.

**Objectives:**

To describe the observed effects of CBD use during treatment with electroconvulsive therapy (ECT).

**Methods:**

To describe in detail the clinical case and to correlate the changes observed in the seizures and the electroencephalogram (EEG) of the ECT sessions when the patient performed concomitant consumption of CBD, in addition to the changes at the clinical level that he experienced during this period.

**Results:**

The subject is a 47-year-old man who required admission for a psychotic decompensation of his schizoaffective disorder bipolar subtype. In recent years, he had no adherence to psychopharmacological treatment or follow-up, and reported self-medicating with CBD cigarettes in varying amounts (he reported 5 to 15 units/day). During admission, pharmacological treatment was instituted with poor response and an acute course of ECT was performed due to a history of good response in a previous episode 10 years ago and the patient’s preference. He remained abstinent from CBD during hospitalization and it was agreed to remain non-consuming at discharge. He presented progressive improvement in the clinical signs from the 4th session, with remission in the 7th session. Upon discharge, it was agreed to perform consolidation ECT on an outpatient basis, initially with weekly sessions and then every two weeks.

From the 2nd outpatient session, a worsening of motor and electroencephalographic seizures was observed in the ECT sessions. A possible relapse into CBD use, which the patient denied, was explored. After the 3rd outpatient session, the patient recognizes relapse into CBD consumption with occasional consumption. During the following 2 sessions, the deterioration in the quality of the EEG pattern progresses and it is decided to interrupt ECT treatment due to the absence of seizures in EEG recording, coinciding with an increase in the daily amount of CBD consumed.

**Conclusions:**

There is little literature on the management of the effects of CBD in ECT treatment. The observations in this clinical case provide valuable information about the combination of CBD and ECT that can be useful to other professionals with similar cases.

**Disclosure of Interest:**

None Declared

